# CXCL5 secreted from macrophages during cold exposure mediates white adipose tissue browning

**DOI:** 10.1016/j.jlr.2021.100117

**Published:** 2021-09-16

**Authors:** Dabin Lee, Dong Wook Kim, Sanghyuk Yoon, A-Reum Nam, Kang-Hoon Lee, Ki-Hoan Nam, Sang-Mi Cho, Yeodae Yoon, Je-Yoel Cho

**Affiliations:** 1 Department of Biochemistry, BK21 PLUS Program for Creative Veterinary Science Research and Research Institute for Veterinary Science, College of Veterinary Medicine, Seoul National University, Seoul, South Korea; 2 Laboratory Animal Resource Center, Korea Research Institution of Bioscience and Biotechnology (KRIBB), Chungju, South Korea

**Keywords:** iWAT, KO mouse, UCP1, M1 macrophage, beta-adrenergic signaling, M1 macrophages, cold stress, thermogenesis, proteomics, BAT, brown adipose tissue, BMDM, bone-marrow-derived monocyte, BW, body weight, C/EBPβ, CCAAT-enhancer-binding protein β, CIDEA, cell-death-inducing DFFA-like effector A, COX8b, cytochrome c oxidase subunit 8B, CXCL5, C-X-C motif chemokine ligand 5, EAR2, eosinophil cationic protein 2, EBF2, early B cell factor 2, ELOVL3, elongation of very long chain fatty acids protein 3, FASP, filter-aided sample preparation, iWAT, inguinal white adipose tissue, KO, knockout, MGL1, macrophage galactose-type lectin-1, p-CREB, phosphorylated cAMP response element-binding protein, PKA, protein kinase cAMP-dependent, PGC1α, peroxisome proliferator-activated receptor gamma coactivator 1-alpha, PPARγ, peroxisome proliferator-activated receptor gamma, PRDM16, PR/SET domain 16, SIRT1, sirtuin1, TNFα, tumor necrosis factor-α, TW, tissue weight, UCP1, uncoupling protein 1, WT, wild type

## Abstract

Adipose tissue affects metabolic-related diseases because it consists of various cell types involved in fat metabolism and adipokine release. CXC ligand 5 (CXCL5) is a member of the CXC chemokine family and is highly expressed by macrophages in white adipose tissue (WAT). In this study, we generated and investigated the function of CXCL5 in knockout (KO) mice using CRISPR/Cas9. The male KO mice did not show significant phenotype differences in normal conditions. However, proteomic analysis revealed that many proteins involved in fatty acid beta-oxidation and mitochondrial localization were enriched in the inguinal WAT (iWAT) of *Cxcl5* KO mice. *Cxcl5* KO mice also showed decreased protein and transcript expression of genes associated with thermogenesis, including uncoupling protein 1 (UCP1), a well-known thermogenic gene, and increased expression of genes associated with inflammation. The increase in UCP1 expression in cold conditions was significantly retarded in *Cxcl5* KO mice. Finally, we found that CXCL5 treatment increased the expression of transcription factors that mediate *Ucp1* expression and *Ucp1* itself. Collectively, our data show that *Ucp1* expression is induced in adipocytes by CXCL5, which is secreted upon β-adrenergic stimulation by cold stimulation in M1 macrophages. Our data indicate that CXCL5 plays a crucial role in regulating energy metabolism, particularly upon cold exposure. These results strongly suggest that targeting CXCL5 could be a potential therapeutic strategy for people suffering from disorders affecting energy metabolism.

The global obesity epidemic has been worsening worldwide since the World Health Organization (WHO) declared obesity a major public health problem and global epidemic in 1997 (1). Adipose tissue was previously categorized into white adipose tissue (WAT) and brown adipose tissue (BAT). WAT stores energy in lipid droplets in the form of triglycerides, while BAT uses many mitochondria to dissipate energy and generate heat (2, 3, 4, 5). In addition, WAT becomes similar to BAT through a process called browning when β-adrenergic receptors are stimulated or exposed to cold. This tissue is called beige adipose tissue and originates from WAT but generates heat similar to BAT (6). The expression of uncoupling protein 1 (UCP1) and peroxisome proliferator-activated receptor gamma coactivator 1-alpha (PGC1α), number of mitochondria, and oxidative metabolism are increased in beige adipose tissue (6). Recently, beige adipose tissue has also received attention because of its ability to consume energy and because of its differences from inguinal WAT (iWAT), such as increased mitochondrial activity and expression of UCP1. Since these facts are strongly associated with energy balance, particularly energy expenditure, the need for studies about adipocytes and browning has consistently increased with the ongoing obesity problem.

The browning of iWAT has a great advantage in terms of energy metabolism, and many studies have been conducted to utilize iWAT for the treatment of obesity and diabetes. Exposure to cold temperatures and exercise is known to increase browning (7), and there are several factors that specifically control the process (5, 8, 9). For instance, transcription factors such as peroxisome proliferator-activated receptor gamma (PPARγ) and CCAAT-enhancer-binding proteins (C/EBPs) are essential elements in the transcriptional cascade of adipocyte maturation. Furthermore, these transcription factors are involved in the differentiation of both BAT and WAT (10). Sirtuin1 (SIRT1), PGC1α, PR/SET domain 16 (PRDM16), and early B cell factor 2 (EBF2) have all been shown to be associated with beige adipogenesis and crucial for UCP1 transcription during browning (11, 12, 13, 14, 15, 16). Exposure to cold is associated with the browning process by increased noradrenaline release and stimulation of various subtypes of β-adrenergic receptors (ADRBs). As a result, brown adipocytes proliferate, and thermogenesis is activated. (17). In contrast, GABA signaling activation is known to exert opposite effects in adipocytes. The constitutive activation of the GABA-BR1 receptor in obese mice causes low UCP1 expression, inducing mitochondrial calcium overload and oxidative stress. Moreover, in experimental animals, a high-fat diet leads to a chronic upregulation of GABA-BR1 in adipose tissue (18).

Adipokines and cytokines such as C-X-C motif chemokine ligand 14 (CXCL14) and fibroblast growth factor 21 (FGF21) are secreted from WAT or immune cells to maintain the homeostasis of fat metabolism. Moreover, these proteins are also involved in the browning process (3, 19, 20, 21). However, their source and functions in adipogenesis are diverse, e.g., adipocytes secrete CXCL14 to recruit M2 macrophages; FGF21 induces PGC1α to cause thermogenesis by enhancing UCP1 expression. CXCL5 is an adipokine secreted from subcutaneous WAT resident-M1 macrophages (22). CXCL5 is known to be an inflammatory cytokine that is highly secreted in the conditions of obesity, diabetes, and infection (22, 23, 24, 25, 26). The roles of CXCL5 in obesity may involve causing inflammation and mediating insulin resistance (22). On the other hand, CXCL5 increases ABCA1 expression in macrophages, thereby increasing cholesterol efflux and inhibiting foam cell formation in atherosclerosis (27). Thus, although a number of studies have shown the involvement of CXCL5 in metabolic disease, the precise role of CXCL5 in obesity-related diseases remains unclear. In this study, we used proteomic analysis with iWAT in both wild-type (WT) and *Cxcl5* knockout (KO) mice to demonstrate the function of CXCL5 in adipose tissue and its browning after cold exposure.

## Materials and methods

### Generation of *Cxcl5* KO mice and animal experiments

*Cxcl5* KO mice were generated using CRISPR/Cas9 system with the sgRNA sequence 5′ CATCTCGCCATTCATGCGGATGG 3′ on C57BL/6N-Tac background mice by the Korea Mouse Phenotyping Center (KMPC). *Cxcl5* KO mice were created by deleting a 16-bp sequence of exon 1 of the *Cxcl5* gene. Mutation of the *Cxcl5* gene was confirmed with WT allele- and mutant allele- specific primers (as listed in supplemental Table S1). All the phenotyping was performed by KMPC following the ARRIVE guidelines. For the cold exposure experiment, 8-week-old male mice were maintained at thermoneutral temperature (30°C) for 3 days before the 1 day (24 h)-long cold exposure (6°C) (28). An injection of CL 316,243 (Cayman) (1.0 mg/kg body weight) was administered intraperitoneally for 3 days at the same time each day. The body weight of each mouse was measured every week, and the body composition was measured in 21-week-old mice. All animal experiments and protocols were approved by Seoul National University Institutional Animal Care and Use Committee (IACUC) (SNU-160825-2-1).

### Cell culture

Bone-marrow-derived monocytes (BMDMs) were isolated from the femurs and tibias of 8- and 9-week-old C57BL/6N-Tac WT mice and *Cxcl5* KO mice. Isolated BMDMs were grown for 7 days in Dulbecco’s Modified Eagle Medium (DMEM) supplemented with 10% FBS, 1% penicillin/streptomycin (P/S), and M-CSF 10 ng/ml. On day 8, to differentiate M0 into M1 and M2 macrophages, the cells were treated with 100 ng/ml LPS (L2654, Sigma) or 20 ng/ml IL-4 (200-04, Peprotech) and 10 ng/ml M-CSF (576402, Biolegend) 24 h. Then, 3T3-L1 preadipocytes were maintained in DMEM supplemented with 10% bovine serum and 1% P/S. To differentiate mature adipocytes, 100% confluent 3T3-L1 cells were cultured in 10% FBS and1% P/S medium supplemented with 0.5 mM IBMX (I-7018, Sigma), 1 μM dexamethasone (D-4902, Sigma, US), and 10 μg/ml insulin (I-6634, Sigma). Two days after the induction of differentiation, the DMEM with 10% FBS supplemented only with 10 μg/ml insulin was replaced. On day 4, only the DMEM with 10% FBS was treated, and the medium was changed once every 2 days.

### Proteomics sample preparation

iWAT was isolated from 21-week-old C57BL/6N-Tac WT and *Cxcl5* KO mice (n = 6) to obtain 80–100 ug of peptides according to the filter-aided sample preparation (FASP) digestion from the study by Sielaff *et al.* (29). The iWAT samples were homogenized with sodium dodecyl sulfate buffer (4% SDS, 100 mM Tris/HCl, pH 7.6). The protein obtained by lysing the tissue was reduced to 0.1 M with DTT for 1 h at 60 °C. After quantifying the protein, it was transferred to a 30-kDa cutoff filter and digested according to the protocol previously described by Sielaff *et al.* (29). Digestion was performed in 50 mM ABC buffer at 37°C overnight with Pierce MS-grade trypsin (trypsin to protein ratio 1:50). Six individual mice were used as biological replicates per group. Then, the two samples were pooled to reduce the variance between individual samples.

Peptides were extracted from six mice in each group, and two mice were pooled to proceed with TMT 6 plex (90066, Thermo Scientific) labeling. TMT labeling was performed according to the manufacturer's protocol, and the labeled peptides were dried with speedvac. Then, SDB-RPS 3 fractionation was performed to identify more proteins. The SDB-RPS stage tip was made by mounting 4 SDB-RPS discs (2241, 3M) in a 200-μl tip with an 18-gauge needle. Each tip was activated with 100 μl of 100% methanol and centrifuged at 1,000 *g* for 2 min. After that, the tip was equilibrated with 1% trifluoroacetic acid (TFA) in water. After dissolving the dried peptide with 1% TFA in water, 90 ug was loaded. Peptides were washed with 100 μl of 0.2% TFA in water and eluted with three buffers of different compositions. For the detailed buffer composition, refer to Mann *et al.* (30).

### LC-MS/MS analysis

Liquid chromatography–tandem mass spectrometry (LC-MS/MS) analysis was performed with Orbitrap Fusion Lumos (IQLAAEGAAPFADBMBHQ, Thermo Scientific) and EASY-nLC 1200 (LC140, Thermo Scientific). An autosampler was used to load 10-μl aliquots of the peptide solutions into an EASY column (Acclaim PepMap™ 100 with i.d. of 75 μm, length of 2 cm, and particle size of 3 μm; 164946, Thermo Scientific). Then, the trapped peptides were separated on an EASY-Spray Column (C18 analytic-column with i.d. of 75 μm, length of 500 mm, and particle size of 2 μm, 100 Å; ES803A, Thermo Scientific). The mobile phases were composed of 100% water (A) and 100% acetonitrile (ACN) (B), and each contained 0.1% formic acid. The LC gradient was initiated with 5% B, increased to 8% B over 1 min, 10% B over 16 min, 40% B over 79 min, and then maintained at 80% B for 9 min and 2% B for an additional 15 min at a flow rate of 250 nl/min. During the chromatographic separation, the Orbitrap Fusion Lumos was operated in a data-dependent acquisition mode. Survey full scans were acquired on a mass range of 400–1,600 m/z with a maximum injection time of 100 ms, an automatic gain control (AGC) target of 2e5 ions, and a resolution of 120,000 and analyzed using the Orbitrap. MS/MS precursors were selected from top n intense ions in 3 s between survey scans, which were fragmented by 37.5% HCD collision energy. MS/MS was acquired with a maximum injection time of 54 ms, an AGC of 5e4 ions, and a resolution of 30,000 and analyzed using the Orbitrap. Previously fragmented precursors were excluded for 30 s. No technical replicates were performed since the analysis was performed after isobaric labeling.

### MS data processing and bioinformatics analysis

The raw data were processed with MaxQuant software (version 1.5.8.3), and MS/MS spectra were searched with the Andromeda search engine against the Mus musculus UniProt database (downloaded on June 29, 2018) at default settings with the minimum number of amino acids ≥6 and unique peptides ≥2. Mass tolerance was set to 4.5 ppm for precursor ions, and the fragment mass tolerance was 20 ppm. Trypsin (cleavage C-terminal to Lys and Arg) was set to enzyme specificity, and a maximum of two missed cleavages were accepted. Carbamidomethyl on Cys was set as a fixed modification, and oxidation on Met and protein N-terminal acetylation were set as variable modifications. The false discovery rate was set to 1% using a target-decoy-based strategy. Output files generated from Maxquant were subjected to Perseus (version 1.6.2.2) to perform bioinformatics analyses.

### RNA isolation and quantitative RT-PCR (qRT-PCR)

RNA was isolated from both cells and tissues with Trizol (15596018, Ambion). In particular, when extracting RNA from iWAT, we referred to the study by Cirera *et al.*, to extract RNA with the maximum amount of fat removed (31). One microgram of purely isolated RNA was reverse-transcribed into cDNA with Omni script (205113, QIAGEN, DE) according to the manufacturer’s protocol. Real-time PCRs (CFX Connect 1855201, BIORAD) were performed using SYBR green (S7563, Invitrogen) and GO taq (M8298, Promega) with specific primers (as listed in supplemental Table S1). The 36B4 gene was used to normalize expressions of all target genes.

### Western blot, ELISA, and histological analysis

For the Western blot analysis, 4% SDS Tris/Hcl pH lysis buffer was used to extract protein from frozen tissues. After the tissues were homogeneously crushed using liquid nitrogen, a lysis buffer was added, and the mixture was left at room temperature for a short period; then, it was boiled at 95°C for 5 min. Then, the cell lysate was centrifuged at 14,000 *g* for 10 min. The protein concentration was determined by a BCA assay, and the same amount of it was loaded on sodium dodecyl sulphate–polyacrylamide gel electrophoresis (SDS-PAGE). Polyvinylidene fluoride (PVDF) was used as a transfer membrane, and after leaving it overnight at 4°C with the primary antibody, it was incubated with the secondary antibody suitable for the primary antibody for 1 h at room temperature. The antibody information is as follows: UCP1 (ab10983, Abcam, UK), phospho-CREB (9198, Cell signaling), and α-tubulin (LF-MA0117, AB Frontier, KR). The CXCL5 level in iWAT was measured by using a mouse CXCL5 ELISA kit (MX000, R&D) according to the manufacturer’s protocol. For histological analysis, iWAT tissues were fixed in the 4% paraformaldehyde for 24 h or more and embedded in paraffin. The deparaffinized and dehydrated sections were stained with hematoxylin and eosin, and they were visualized under a light microscope (ECHO). The average of sizes and the number of lipid droplets were calculated with ImageJ (NIH).

### Mitochondrial membrane potential assay and MitoTracker labeling

The mitochondrial membrane potential (ΔΨ_m_) was measured by JC-1 (ab113850, Abcam, UK). Differentiated 3T3-L1 cells were treated with 5-μm JC-1 for 20 min at 37°C. Then, the cells were washed with PBS. To label the mitochondria, 50 nM of MitoTracker (M-7510, Thermo Scientific) in media without FBS was added to cells, and they were incubated at 37°C for 1 h. The cells were then washed with PBS and fixed with 4% paraformaldehyde for 10 min at room temperature. They were observed with a fluorescence microscope (ECHO).

### Statistical analysis

All the analyses were performed with Graph Pad Prism 7, and the data are presented as the mean ± standard error of the mean (SEM). The statistical significance of data was determined by unpaired two-tailed Student’s *t* test and one-way ANOVA and two-way ANOVA for comparisons with multiple variables. Significance was defined as *∗P* < 0.05, *∗∗P* < 0.01, *∗∗∗P* < 0.001 and *∗∗∗∗P* < 0.0001.

## Results

### CXCL5 is a cold-inducible chemokine in iWAT

**Fig. 1:**
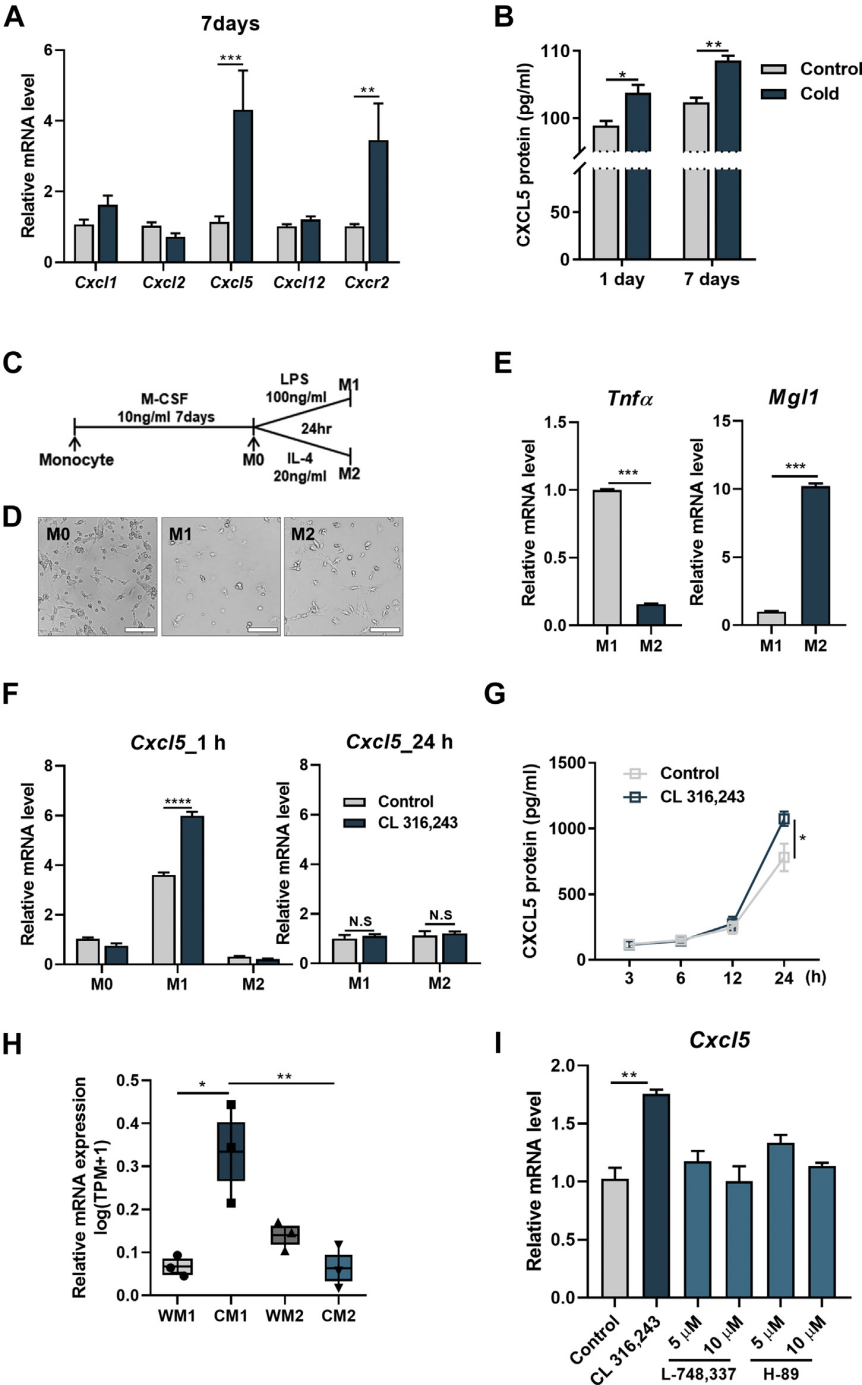
M1 macrophages express *Cxcl5* under cold exposure or β3-adrenergic receptor activation in iWAT. A: mRNA expression of chemokines in iWAT after 3 days in thermoneutral conditions (30°C) and then in cold conditions (6°C) for 17 days (n = 3–5). B: ELISA analysis of CXCL5 protein expression in iWAT after 3 days in thermoneutral conditions (30°C) and then in cold conditions (6°C) for 1 or 7 days. C: Simple schematic diagram of primary macrophage differentiation. BMDMs isolated from mice were treated with M-CSF at a concentration of 10 ng/ml for 7 days and then treated with 100 ng/ml LPS to differentiate into M1 macrophages and 20 ng/ml IL-4 to differentiate into M2 macrophages. Differentiated M1 macrophages were treated with CL 316,243 at a concentration of 1 μM. D: Micrographs of M0, M1, and M2 macrophages (Scale bar = 100 μm) and (E) mRNA expression of M1 and M2 macrophage markers. F: mRNA expression of *Cxcl5* when treated with 1 μM CL 316,243 for 1 h and 24 h. G: Protein concentration of CXCL5 in M1 macrophages in time course treatment with 1 μM CL 316,243 as determined by ELISA. H: Expression of *Cxcl5* in M1 and M2 macrophages of visceral adipose tissue (VAT) RNA-sequencing data during cold exposure. I: Primary M1 macrophages were pre-incubated a β3-adrenoceptor antagonist (5, 10 μM) or PKA inhibitor (5, 10 μM) 1 h before treatment with 1 μM CL 316,243 for 2 h. Data are presented as the mean ± SEM. *∗P* < 0.05, *∗∗P* < 0.01, *∗∗∗P* < 0.001, and *∗∗∗∗P* < 0.0001 for the effects of cold exposure or CL 316,243 treatment compared with controls by two-tailed Student’s *t* test (A–E, I) and two-way ANOVA (F–H).

Various chemokine receptors have been studied in the development of adipocytes and their differentiation process. These chemokines include CXCR7 in preadipocytes, CXCR2 in adipocytes, and CXCR4 in BAT (32, 33). To determine cold-responsive chemokines in the iWAT of mice, we mainly focused on the ligand chemokines of CXCR2 such as CXCL1, CXCL2, CXCL5, and CXCL12 (Fig. 1A). When the mice were exposed to cold conditions for 7 days, only CXCL5 drastically increased, while the other chemokines did not change (Fig. 1A). The increase in CXCL5 in a cold setting was confirmed by protein level with ELISA. Both 1-day and 7-day cold exposures significantly increased CXCL5 chemokine protein expression in iWAT when compared with the control (Fig. 1B). Adipose tissue consists of diverse cell types, including preadipocytes, fibroblasts, vascular endothelial cells, and immune cells, and CXCL5 is secreted from M1 macrophages. Hence, we identified the source of cells secreting CXCL5 in cold conditions (22). We isolated BMDM cells to differentiate them into M0, M1, and M2 macrophages (Fig. 1C, D). Macrophage differentiation was confirmed by the expression of two inflammatory cytokines. The expression pattern of tumor necrosis factor alpha (*Tnfα*), which is known to be high only in M1 macrophages, and *Mgl1*, which is known to be high only in M2 macrophages, showed that the two subtypes of macrophages were well differentiated (Fig. 1E). Subsequently, to test whether macrophages can respond to cold exposure and CL 316,243, a β-adrenergic agonist, we first confirmed the expression of *Adrb3* in M1 macrophages differentiated from M0 cells (supplemental Fig. S1A) and in the public transcriptome data published by Chang *et al.* (supplemental Fig. S1B) (34). In the M1 macrophages, *Cxcl5* transcription was significantly increased by CL 316,243 treatment for 1 h but then disappeared in 24 h (Fig. 1F). However, this early response of CXCL5 protein levels to cold was observed at 24 h exposure (Fig. 1G). On the other hand, when M0 macrophages were stimulated with IL-4 to differentiate into M2 macrophages, M2 macrophages did not react with CL 316,243 for *Cxcl5* expression (Fig. 1F). Using a previously reported high-throughput sequencing dataset (34), we verified our results that cold exposure triggers CXCL5 secretion from M1 macrophages. In the previous study, they sorted out M1 and M2 macrophages using FACS from adipose tissues in mice that were adapted at 18°C for 7 days and subsequently exposed to 4°C for 8 days. The result clearly shows that *Cxcl5* is expressed at the highest level in M1 macrophages when exposed to cold (Fig. 1H). Next, we further confirmed that the signaling pathway associated with *Cxcl5* expression is triggered by CL 316,243 treatment. As CL 316,243 is known to activate the β3-adrenergic receptor signal, we treated with a β3-adrenoceptor antagonist, L-748,337, and a PKA signal inhibitor, H-89, before the CL 316,243 treatment. The increase in *Cxcl5* by CL 316,243 was completely blocked by L-748,337 and H-89 (Fig. 1I). Thus, *Cxcl5* expression was regulated via the β3-adrenergic receptor signal and PKA signaling pathway in M1 macrophages treated with CL 316,243.

### Generation of *Cxcl5* KO mice by CRISPR/Cas9

**Fig. 2:**
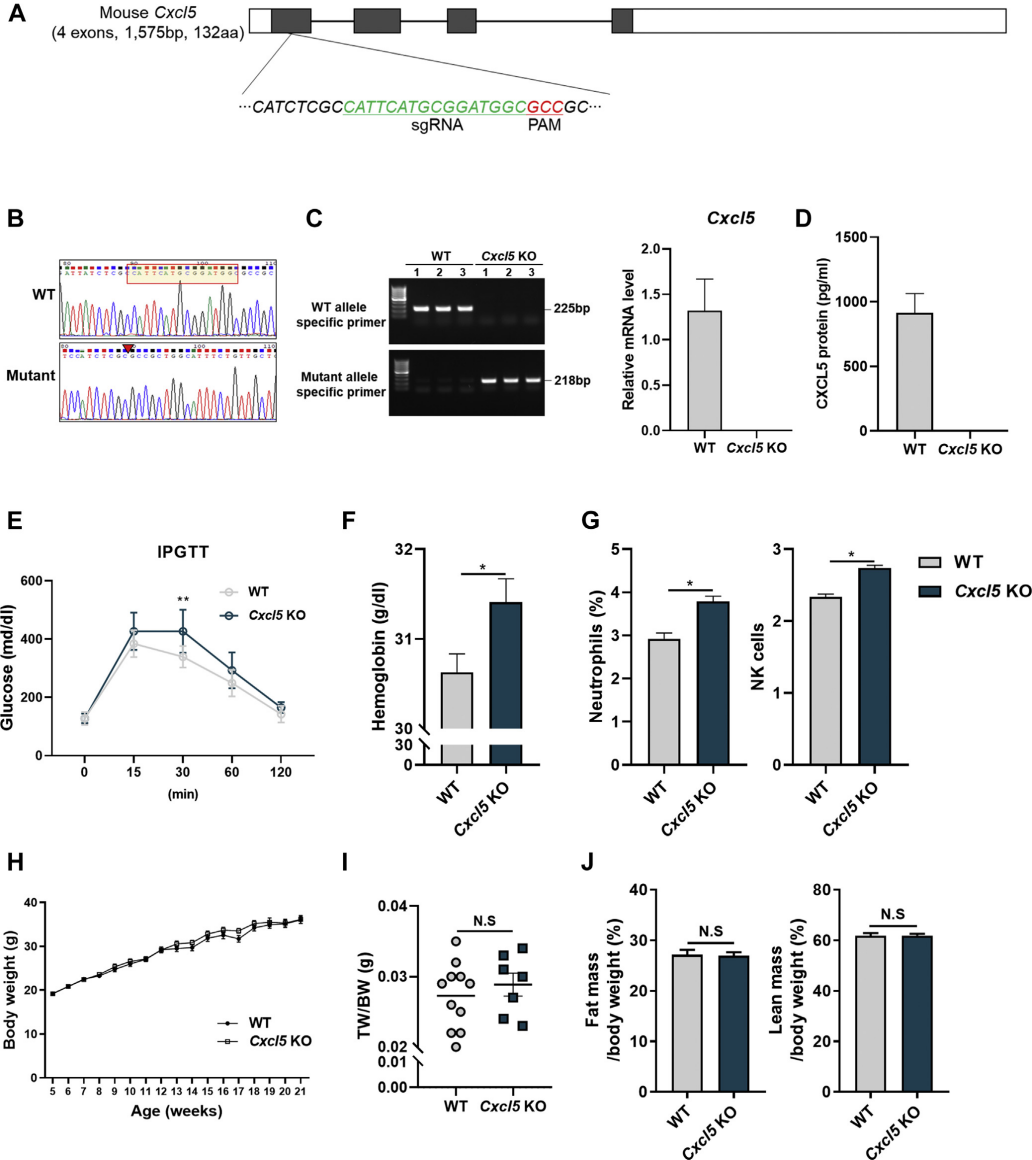
*Cxcl5* KO by CRISPR/Cas9 system and basic phenotype. A: Schematic illustration of the *Cxcl5* locus. A 16-bp sequence was deleted with sgRNA and the PAM sequence located in exon 1. B: Sanger sequencing data confirmed the deletion site in the mutant allele. C: Confirmation of *Cxcl5* KO by performing conventional PCR with WT- and mutant-specific primers, respectively. D: CXCL5 ELISA data in the serum of WT and *Cxcl5* KO mice. E: Blood glucose levels during the intraperitoneal glucose tolerance test (IPGTT) in 13-week-old WT (n = 9) and *Cxcl5* KO (n = 9). F: Amount of hemoglobin in WT (n = 9) and *Cxcl5* KO (n = 9) mice by Hematology analysis. G: Neutrophil and NK cell counts in the splenocytes of WT (n = 3) and *Cxcl5* KO (n = 3) mice. H: Body weight from 5 to 21 weeks for WT (n = 13) and *Cxcl5* KO (n = 11) mice. I: iWAT weight/body weight of WT and *Cxcl5* KO mice. J: Body composition in WT and *Cxcl5* KO mice. Data are expressed as the mean ± SEM. Statistical analysis was performed using two-tailed unpaired Student's *t*-tests. *∗P* < 0.05 and *∗∗P* < 0.01.

The *Cxcl5* KO mice were generated using the CRISPR/Cas9 system. We designed guide RNAs targeting the first exon of the CXCL5 locus, which was selected by the most efficient sgRNA binding with minimum off-target effects, to generate homozygous *Cxcl5* KO mice (Fig. 2A). A Cas9 and sg RNA system led to a 16-bp (CATTCATGCGGATGGC) deletion mutation on the first exon of the *Cxcl5* locus (Mus musculus chromosome 5: 81,321-81,336), which caused a frameshift mutation that generated a premature stop codon after 45 bp. As a result, while WT CXCL5 encodes 132 amino acids including the conserved C-X-C motif (from 41 to 132), the mutant CXCL5 only retained 17 original amino acids with 15 extra abnormal amino acids not present in the mouse genome and protein sequence. *Cxcl5* KO was confirmed by genomic DNA sequencing and conventional PCR and qRT-PCR with WT and mutant allele-specific primers (Fig. 2B, C). We next confirmed that *Cxcl5* KO mice were successfully generated by showing the completely depleted protein expression of CXCL5 in KO mice using ELISA (Fig. 2D).

As a chemokine, *Cxcl5* KO has some differences in its immunologic features, such as hemoglobin in blood and changes in neutrophils and natural killer (NK) cells in splenocytes, when compared with WT in normal conditions (Fig. 2E, F). Except glucose tolerance, there were no observable physical or biochemical differences found in homozygous *Cxcl5* KO male mice when compared with WT mice. For instance, body weight, tissue weight, body composition, blood insulin concentration, grip strength, and pathology were not significantly different between *Cxcl5* KO and WT mice (Fig. 2G–I, supplemental Fig. S2, supplemental Table S2–S5). These data support the changes in CXCL5 and the effects associated with cold exposure and β3-adrenergic stimulation seen in Fig. 1.

### Proteome analysis revealed that CXCL5 deficiency alters diverse molecular phenotypes including mitochondrial activity

**Fig. 3:**
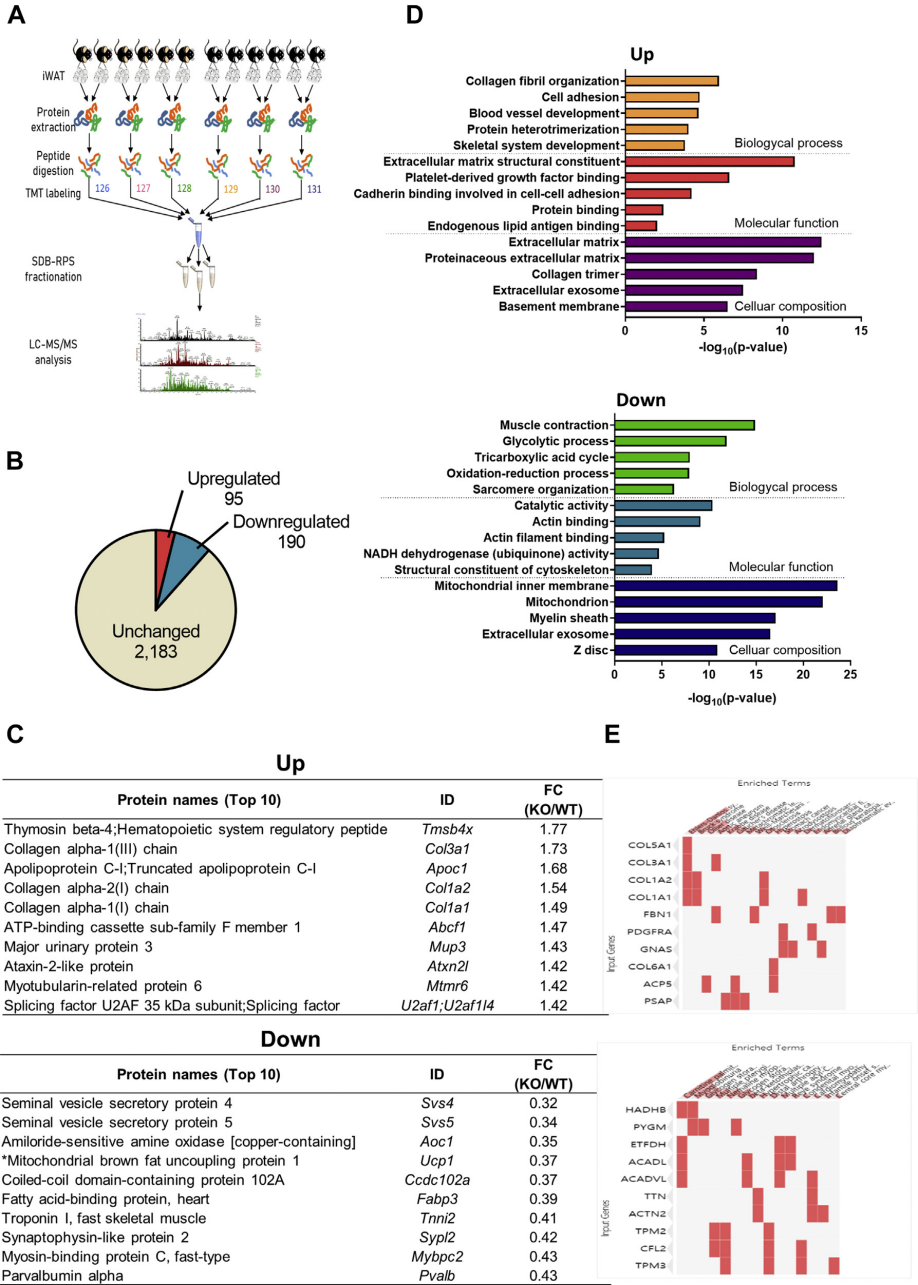
Proteomic analysis revealed that *Cxcl5* KO alters diverse molecular pathways including mitochondrial activity. A: Schematic diagram of iWAT proteomics. iWAT was isolated and homogenized from 21-week-old mice for protein extraction. Proteins were digested into peptides using the filter-aided sample preparation (FASP) method. After performing TMT labeling, peptides were pooled two by two for LC-MS/MS analysis. B: Total number of ID proteins and number of proteins up- or downregulated in *Cxcl5* KO mice. C: Top 10 up- and downregulated proteins in *Cxcl5* KO mice. D: GO analysis of up- and downregulated proteins in *Cxcl5* KO. E: GO analysis in the Jansen Disease database.

Quantitative proteomic analysis was performed using LC-MS/MS in iWAT obtained from both WT and *Cxcl5* KO mice. Figure 3A briefly illustrates the process of proteome analysis. After tissue sampling, protein isolation and digestion were performed, and 6-plex TMT labeling and SDB-RPS fractionation were performed for LC-MS/MS analysis. The detailed procedure is described in the Methods section. A total of 2,468 proteins, including 95 upregulated and 190 downregulated proteins, were isolated in *Cxcl5* KO mice (Fig. 3B, supplemental Table S6). The top 10 up- and downregulated proteins in *Cxcl5* KO mice are listed in Fig. 3C. Unexpectedly, several types of collagens were upregulated in the iWAT of *Cxcl5* KO mice. Instead, mitochondrial brown fat UCP1, which has been well-characterized among cold-responsive target proteins, was significantly downregulated (Fig. 3C). Gene ontology (GO) and pathway enrichment analysis using upregulated genes revealed that the increased proteins in *Cxcl5* KO mice enriched the terms for cell adhesion, cellular response to lipid, and energy reverse metabolism. In contrast, the list of proteins downregulated in *Cxcl5* KO mice was remarkably enriched in many mitochondrial-related terms (Fig. 3D). Furthermore, the downregulated proteins in *Cxcl5* KO mice retrieved terms related to metabolic pathways such as glycolytic process, oxidation–reduction process, and the TCA cycle in GO biological processes. Notably, in terms of disease, there are several common proteins such as hydroxyacyl-CoA dehydrogenase trifunctional multienzyme complex subunit beta (HADHB), electron transfer flavoprotein dehydrogenase (ETFDH), Acyl-CoA dehydrogenase long chain (ACADL), and Acyl-CoA dehydrogenase very long chain (ACADVL) shared by the downregulated proteins in *Cxcl5* KO mice and proteins that are associated with carnitine palmitoyltransferase II deficiency. This deficiency is a metabolic disorder characterized by an enzymatic defect that prevents long-chain fatty acids from being transported into the mitochondria for utilization as an energy source (Fig. 3E). Altogether, the proteomic analysis revealed that CXCL5 is functionally involved in several cellular metabolic processes via its influence on mitochondrial activity and processing of collagen protein.

### CXCL5 plays a crucial role in mitochondrial function in iWAT

**Fig. 4:**
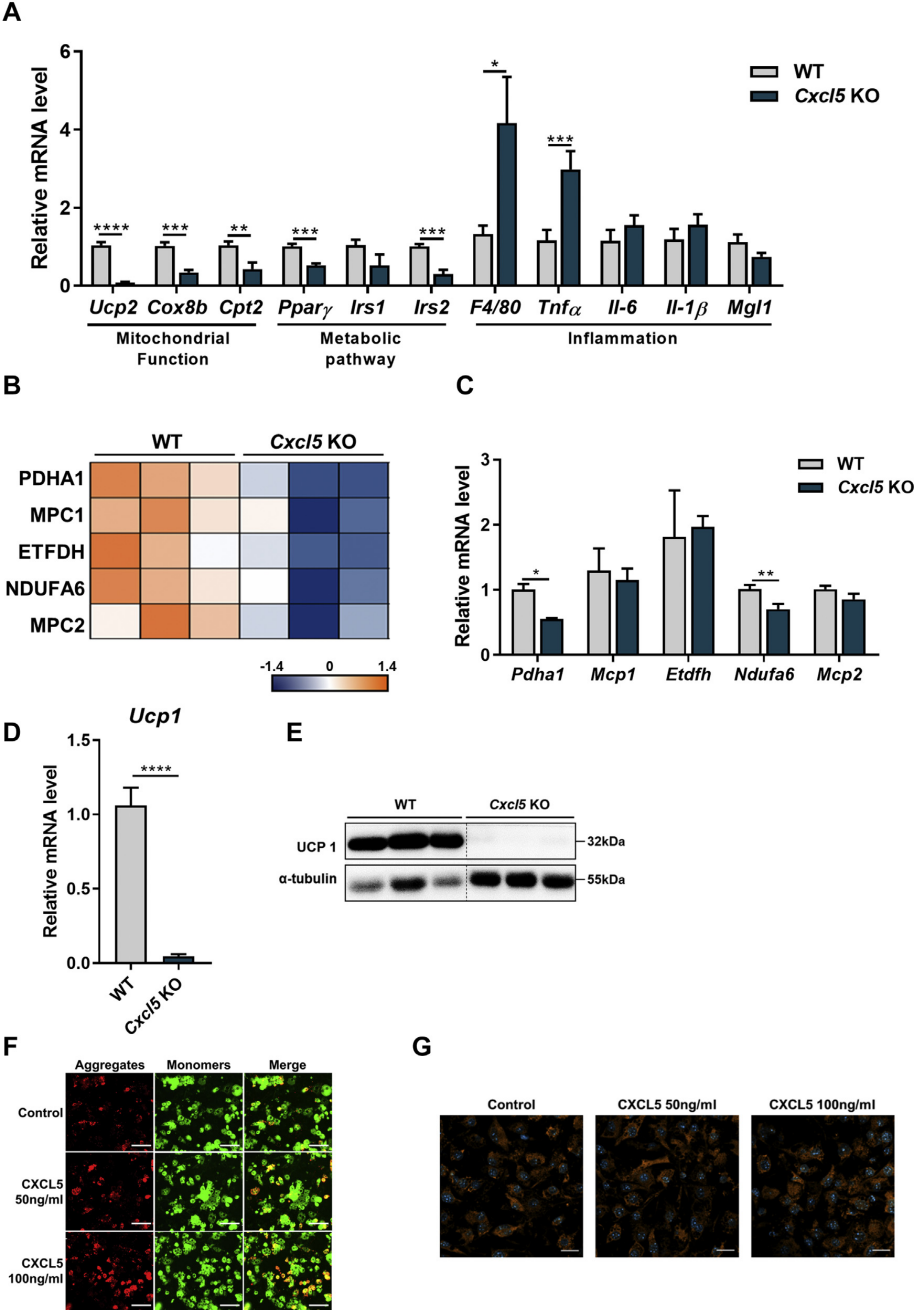
Downregulation of mitochondrial and metabolic pathway genes in *Cxcl5* KO mice. A: mRNA expression of mitochondrial, metabolic pathway, and inflammatory genes in *Cxcl5* KO mice. B: Heat map of mitochondrial-related proteins in proteomics data and (C) qRT-PCR. D: Suppressed *Ucp1* expression at the RNA level and (E) at the protein level in *Cxcl5* KO mice. Western blot data was spliced to arrange the phenotype. The spliced position was indicated by vertical dashed line. F: Mitochondrial membrane potential as measured by JC-1 staining and (G) MitoTracker staining in control and administration of recombinant CXCL5 (50, 100 ng/ml). Red: JC-1 aggregate, green: JC-1 monomer. Scale bar = 100 μm, Blue shows nuclei staining with Hoechst 33342. Data are expressed as the mean ± SEM. Statistical analysis was performed using two-tailed unpaired Student's *t*-tests. *∗P* < 0.05, *∗∗P* < 0.01, *∗∗∗P* < 0.001, and *∗∗∗∗P* < 0.0001.

To investigate the putative function of CXCL5 in mitochondrial activity, we first focused on the list of genes associated with mitochondrial function and metabolic pathways instead of inflammatory genes. As a result of the proteome analysis, representative gene transcriptions of both mitochondrial function (*Ucp2, Cox8b,* *Cpt2*) and metabolic pathway (*Pparγ, Irs1,* and *Irs2*) were significantly downregulated in *Cxcl5* KO mice (Fig. 4A). This mitochondrial deterioration was also found in our proteome analysis results as shown by a significant decrease in mitochondrial protein expression in CXCL5 deficiency (Fig. 4B). Five mitochondrial proteins, pyruvate dehydrogenase alpha 1 (PDHA1), mitochondrial pyruvate carrier 1 (MPC1), electron transfer flavoprotein dehydrogenase (ETFDH), NADH dehydrogenase [ubiquinone] 1 alpha subcomplex subunit 6 (NDUFA6), and mitochondrial pyruvate carrier 2 (MPC2) were dramatically decreased < 0.1 fold in *Cxcl5* KO mice. However, only PDHA1 and NDUFA6 were decreased at the transcription level (Fig. 4C). Notably, downregulation of UCP1 in *Cxcl5* KO mice was confirmed by qRT-PCR and western blot. UCP1 is a protein located in the mitochondrial inner membrane. Both the RNA (Fig. 4D) and protein (Fig. 4E) levels of UCP1 were extremely lower in *Cxcl5* KO mice than in WT mice. We then directly investigated the changes in mitochondrial membrane potential by CXCL5 treatment. JC-1 staining clearly showed that the mitochondrial membrane potential increased in adipocytes when treated with CXCL5 (Fig. 4F). However, there was no difference in the number of mitochondria when adipocytes were treated with CXCL5 (Fig. 4G). Therefore, in the absence of CXCL5, mitochondrial genes including *Ucp1* and their proteins were decreased; thus, the mitochondrial function decreased in the iWAT of *Cxcl5* KO mice.

### *Cxcl5* KO mice undergo less thermogenic action even in cold exposure and after CL 316,243 treatment

**Fig. 5:**
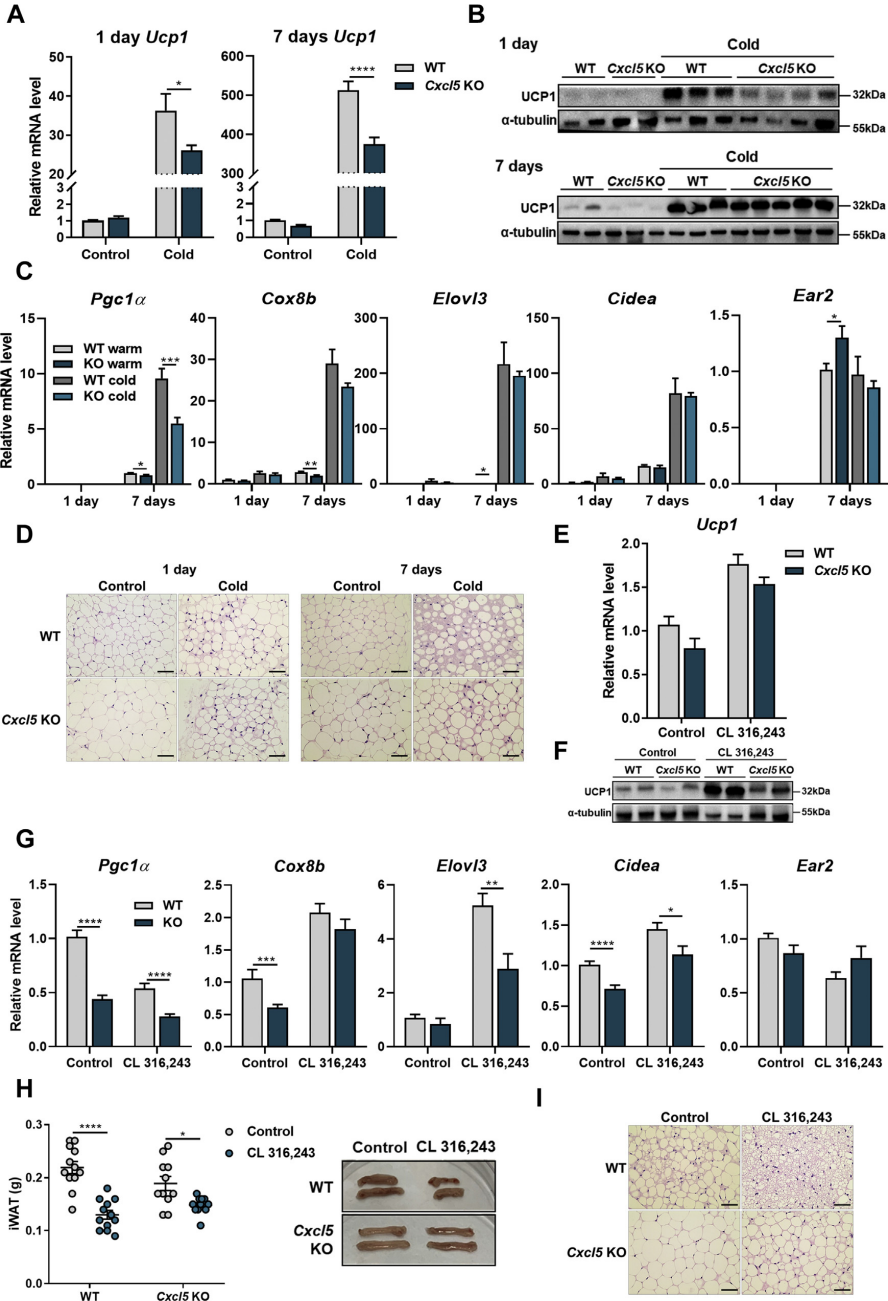
*Cxcl5* KO inhibits browning of WAT. Both WT and *Cxcl5* KO mice were kept at thermoneutral conditions for 3 days followed by 1 day and 7 days of cold exposure. (n = 3–5). A: *Ucp1* expression was measured by qRT-PCR and by (B) Western blot, and *36b4* gene and α-tubulin were used for internal controls for RNA and protein, respectively. C: mRNA expression of *Pgc1α, Cox8b, Elovl3, Cidea*, and *Ear2* as analyzed by qRT-PCR. D: Representative H&E-stained image of iWAT after 1 day and 7 days of cold exposure. Scale bar = 200 μm. E: mRNA and (F) protein expression levels of UCP1 in iWAT after intraperitoneal administration of CL 316,243 (0.5 mg/kg body weight/day) and PBS for 3 days (n = 10–11). G: mRNA expression of *Pgc1α, Cox8b, Elovl3, Cidea*, and *Ear2*. H: iWAT weight in WT and *Cxcl5* KO mice when treated with CL 316,243. Below is an image of iWAT extracted from *Cxcl5* KO and WT mice. I: Representative H&E stained image of iWAT 3 days after CL 316,243 injection. Scale bar = 200 μm. Data are expressed as the mean ± SEM. Statistical analysis was performed using multiple *t*-tests. *∗P* < 0.05, *∗∗P* < 0.01, *∗∗∗P* < 0.001, and *∗∗∗∗P* < 0.0001.

We thus investigated the role of CXCL5 under cold exposure and the activation of β-adrenergic receptor signaling. After housing WT and *Cxcl5* KO mice at thermoneutrality (30°C) for 3 days, we divided them into a control group (30°C) and a cold exposure group (6°C), incubated them for 1 day or 7 days, euthanized them, and isolated iWAT. Interestingly, *Ucp1* transcription was not fully upregulated in the iWAT of cold-exposed *Cxcl5* KO mice (Fig. 5A). Cold stress increased UCP1 expression in both *Cxcl5* KO and WT mice, but the expression level of UCP1 in cold exposure was significantly less in *Cxcl5* KO than in WT mice and was confirmed in the UCP1 protein level. Thermoneutral conditions before cold exposure fully suppressed UCP1 expression in both WT and *Cxcl5* KO mice. After cold exposure, UCP1 protein levels were remarkably induced in WT mice from day 1 but were not fully induced in *Cxcl5* KO mice until day 7 (Fig. 5B). This phenomenon confirms that CXCL5 contributes to the regulation of UCP1 expression in response to cold exposure. Indeed, PGC1α, one of the major regulators of UCP1, significantly responded to 7 days of cold exposure, while the expression levels of four other genes were not different between *Cxcl5* KO and WT mice after cold exposure (Fig. 5C).

Moreover, after both 1 day and 7 days of cold exposure, the histology of *Cxcl5* KO mice showed larger lipid droplets than those in WT mice in warm conditions (control). Cold exposure for 1 day did not lead to a clear difference in the iWAT between WT and *Cxcl5* KO mice. However, there were significant differences in browning after 7 days of cold exposure between WT and *Cxcl5* KO mice. In the iWAT of WT mice, the lipid droplet size became smaller, while the sizes of other structures increased, but no clear changes were found in the iWAT of *Cxcl5* KO mice (Fig. 5D). Overall, CXCL5 regulates the expression of *Ucp1* and thermogenic factors induced by cold exposure and consequently affects fat metabolism.

We thus mimicked the cold exposure condition by directly injecting the β-adrenergic agonist CL 316,243 into mice to confirm the difference between WT and *Cxcl5* KO under cold exposure conditions. Similar to the cold-exposed data, CL 316,243-treated KO mice had a lower increase in *Ucp1* than WT mice, and the difference was reflected in the protein level (Fig. 5E, F). This difference confirmed that the expression levels of genes related to thermogenesis were lower in *Cxcl5* KO mice treated with CL 316,243 (Fig. 5G). We evaluated whether this phenomenon affects the weight and size of tissue when treated with CL 316,243 since it is known that this treatment reduces tissue weight. As a result, the iWAT of WT mice decreased dramatically (*P* < 0.0001), but the iWAT of *Cxcl5* KO mice did not decrease as much (Fig. 5H). In addition, reduced body weight was found only in WT mice but not in *Cxcl5* KO mice (supplemental Fig. S3A). As expected, histological analysis also showed that the lipid droplets in *Cxcl5* KO mice were essentially larger than those in WT mice (Fig. 5I).

### β-adrenergic agonist stimulates the release of CXCL5 from macrophages and stimulates UCP1 expression in adipocytes

**Fig. 6:**
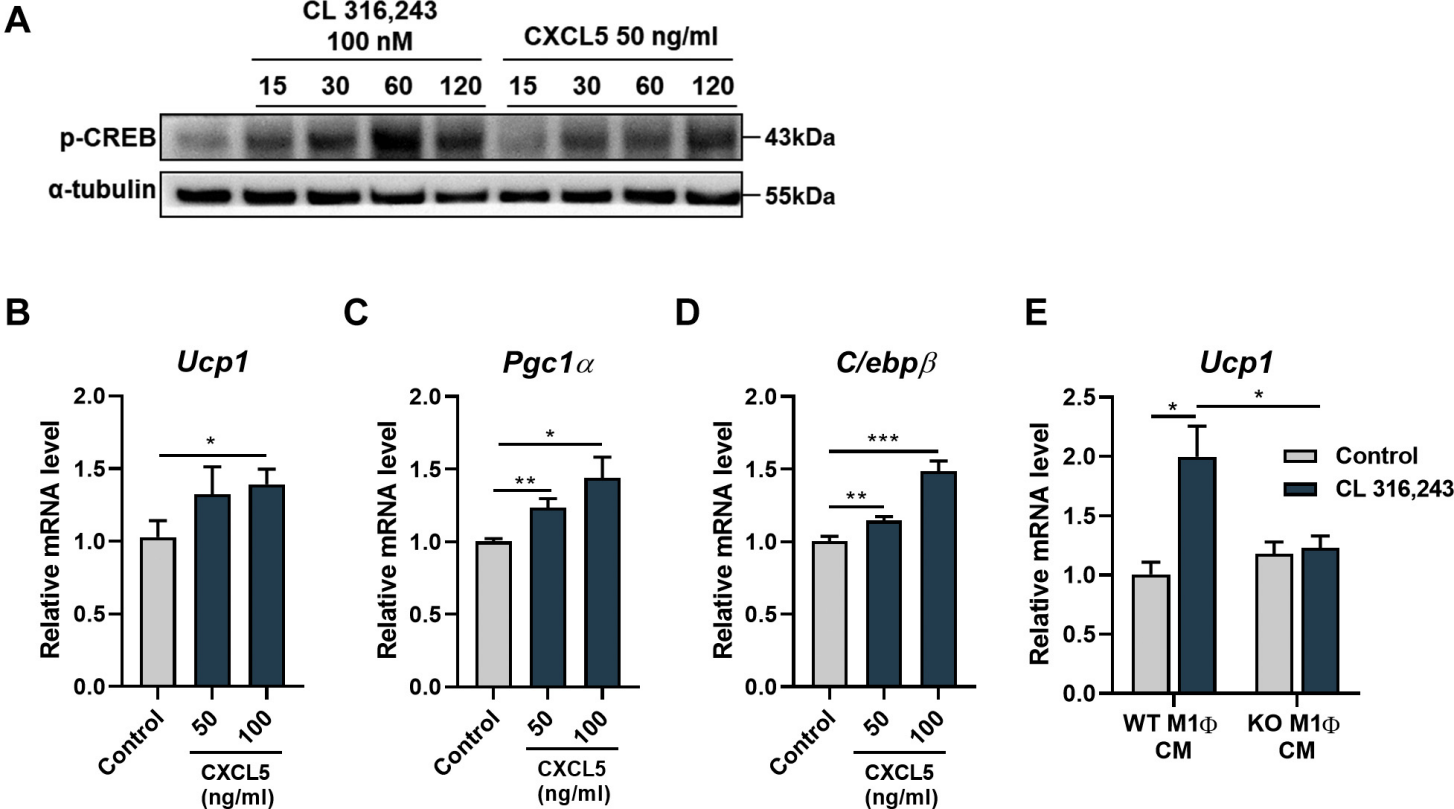
CXCL5 increases *Ucp1, Pgc1α* and *C/ebpβ* expression. A: Time-dependently increased p-CREB level after both CL 316,243 and CXCL5 treatment. A-tubulin was used for an internal control. B: Increased expression of *Ucp1*, together with its transcription factors (C) *Pgc1α* and (D) *C/ebpβ* with the introduction of CXCL5. Data are expressed as the mean ± SEM. Statistical analysis was performed using two-tailed unpaired Student’s *t*-tests. *∗P* < 0.05, *∗∗P* < 0.01, and *∗∗∗P* < 0.001.

We then determined how CXCL5 regulates *Ucp1* expression under cold exposure. Since the canonical β-adrenergic receptor signaling pathways through p-CREB, we first examined p-CREB activation under the CXCL5 treatment condition. As expected, treatment with CL 316,243 in 3T3-L1 cells activated the p-CREB signal. Notably, CXCL5 treatment also activated p-CREB similarly (Fig. 6A). Moreover, *Ucp1* and its major transcription factors, *Pgc1α* and *C/ebpβ*, were continually increased by CXCL5 treatment at the transcriptional level (Fig. 6B–D).

These results allow us to hypothesize that CXCL5 secreted from M1 macrophages within iWAT may triggers *Ucp1* expression in adipocytes, which play a crucial role in the browning of iWAT. Thus, we measured the *Ucp1* expressions levels in 3T3-L1 adipocytes treated with supernatants obtained from M1 macrophage cultures with or without CL 316,243. Importantly, *Ucp1* expression in 3T3-L1 cells was increased in the group of WT-M1-conditioned media treated with CL 316,243. However, KO-M1-conditioned media treated with CL 316,243 did not show elevated *Ucp1* expression in 3T3-L1 cells (Fig. 6E). These data along with the data in Fig. 1 indicate that CXCL5 secretion from M1 macrophages was increased by CL 316,243 and that CXCL5 gives a signal to surrounding adipocytes to induce *Ucp1* expression.

## Discussion

Obesity and diabetes due to poor diet and lifestyle are important health problems worldwide. One of the treatment methods in the spotlight for metabolic diseases is to increase energy expenditure. Energy consumption can be increased in a way that improves mitochondrial function or increases fatty acid oxidation of WAT. Beige adipose tissue can increase energy consumption via thermogenesis due to the increase in proteins such as UCP1 (35, 36). Recently, browning has been studied more frequently as a treatment strategy for metabolic diseases by utilizing the energy consumption of WAT because healthily consuming excess fat through browning is safer than surgery or drug treatment, which may cause negative side effects.

Browning occurs when white adipocytes are exposed to cold; however, recently, chemokines and growth factors are being studied because they also involve in energy consumption. FGF2, one of the most well-known growth factors, is known to enhance not only BAT but also iWAT and fat metabolism (21, 28, 37, 38). In addition, a recent study demonstrated that FGF6 and FGF9 are involved in energy metabolism by regulating the expression of UCP1 in brown fat (38, 39). In addition, chemokines are known to induce both positive and negative effects on energy metabolism. For instance, knockout of CCL2 and CCR7 increases the expression of UCP1 (40, 41), but oppositely, CXCL12 and CXCL14 play positive roles in increasing UCP1 expression and fat metabolism (19, 33). In contrast, it is also known that proinflammatory cytokines interfere with beige adipogenesis. In particular, several studies have reported that M1 macrophages inhibit browning by secreting proinflammatory cytokines such as TNFα and IL-1β in the obese state (42, 43, 44). CXCL5 is also a well-known proinflammatory chemokine (23, 45). However, as shown in this study, the involvement of CXCL5 in metabolic diseases has also been reported. In the serum of diabetic-induced db/db mice and obesity-induced ob/ob mice, the CXCL5 concentration was two times higher than that in lean mice. The CXCL5 concentration was also high in the serum when the notification method was applied to C57 mice for 13 weeks (46). Furthermore, a high CXCL5 concentration in serum is known to affect obesity, hyperglycemia, and islet function (23). Thus, the role of CXCL5 in inflammatory conditions as well as in normal conditions including energy metabolism and browning of iWAT should be better understood.

First, as shown in Fig. 2, *Cxcl5* KO mice were not significantly different from WT mice in their general body phenotypes such as body weight and adipocyte tissue weight (Fig. 2H–J, supplemental Fig. S1A). Instead, as is known, *Cxcl5* KO mice showed some significant differences in their immune systems. For instance, NK cells and neutrophils were significantly increased in the splenocytes of *Cxcl5* KO mice (Fig. 2G). This finding might be correlated with the increased levels of F4/80 and TNFα (Fig. 4A). This discrepancy in the immune system should be characterized further. Moreover, a detailed characterization including in different sexes, hormones, high-fat diet, and cold temperatures, which can considerably influence metabolism and thermogenic biological processes, is needed.

Despite these limitations, our data using *Cxcl5* KO mice are very meaningful to show that CXCL5 plays an important role in metabolism in both normal and cold exposure conditions. Under normal conditions, several genes involved in mitochondrial function and metabolic pathways were decreased in *Cxcl5* KO mice (Fig. 4). In addition, the proteomic analysis revealed that muscle contraction-related proteins and glycolysis-related proteins were decreased in *Cxcl5* KO mice. Recent studies have shown that a subset of beige adipocytes, glycolytic beige fat (g-beige), arises from muscle lineage (47). In addition, beige adipocytes suppress adipose tissue fibrosis (48). These phenomena are very similar to our results shown in Fig. 3C–E, which demonstrates collagen fibril organization-related protein such as collagen fibrils; it is possible that CXCL5 controls g-beige adipocytes, whereas *Cxcl5* KO suppresses the fibrosis program. However, there are some missing or conflicting points to draw conclusions from our experiments. For instance, our experimental conditions were not suitable for the g-beige fat development, and β3-AR expression was lower in *Cxcl5* KO mice than in WT mice, which might indicate a better condition for g-beige fat development. Thus, further characterization of the subtype of beige fat would be interesting.

We then determined the cellular origin of CXCL5. CXCL5 was expressed in M1 macrophages but not in M2 macrophages under cold conditions. This is a clear discrepancy with the findings of a previous study by Cereijo *et al.* (19), who showed the involvement of M2 macrophages recruited by CXCL14 in cold conditions to induce browning of adipocytes. In addition, several review papers have explained that the proinflammatory cytokines secreted by M1 macrophages cause inflammation in adipose tissue, leading to insulin resistance (42, 49). However, in this study, M1 macrophages expressed CXCL5 by adrenergic signaling to induce *Ucp1* expression in iWAT. This may mean that diverse cell types and chemokine signals are involved in iWAT browning.

We also determined the function of CXCL5 in cold exposure. Cells treated with recombinant CXCL5 or with WT-M1 macrophage-conditioned media with CL 316,243 stimulated the expression of *Ucp1* (Figs. 1, 6). In contrast, the expression of *Pgc1α*, a major transcription factor for *Ucp1*, was insufficiently induced in *Cxcl5* KO mice under cold exposure. This phenomenon was clearer under the CL 316,243 treatment condition. In addition to *Pgc1α*, several regulator genes involved in *Ucp1* expression, such as Cox8b, Elovl3, and Cidea, were not as increased in *Cxcl5* KO mice compared with WT mice under cold exposure (Fig. 5G). There is a slight difference between cold exposure and its mimicry induced by CL 316,243 treatment because cold exposure involves complex signaling pathways while CL 316,243 treatment induces only β-adrenergic signaling (50). This result may mean that CXCL5 triggers one of the noncanonical pathways that regulate Ucp1 expression in adipocytes. Furthermore, these results suggest that not only *Ucp1* expression through β-adrenergic receptor signaling in adipocytes under cold conditions but also many other thermogenic factors and complex cellular matrix are important for iWAT browning.

Thus, this study provides sufficient evidence for the source and mechanism of CXCL5 expression and the roles of CXCL5 in activating UCP1 via β-adrenergic receptor signals in cold conditions or after CL 316,243 treatment.

## Data availability

All data regarding to this study are included in supplemental data. Mass spectrometry data have been deposited in the ProteomeXchange Consortium via the jPOST partner repository with the dataset identifier ProteomeXchange: PXD027291 and jPOST: JPST001253.

## Supplemental data

This article contains supplemental data.

## Conflict of interest

The authors declare that they have no conflicts of interest with the contents of this article.
