# Supplementary material for: CXCL5 secreted from macrophages during cold exposure mediates white adipose tissue browning
Source: J Lipid Res. 2021 Sep 16;62:100117. doi: 10.1016/j.jlr.2021.100117 (PMC8512628; doi:10.1016/j.jlr.2021.100117)
Supplement: Supplemental Tables S1, S3, and S5 and Supplemental Figures S1–S3 [file mmc1.pdf]

Table S1.Conventional/qRT-PCR primer

Gene name	Forward sequence (5' → 3')	Reverse sequence (5' → 3')
**WT primer**	ATCTCGCCATTCATGCGGAT	TTGCGGCTATGACTGAGGAAG
**Cxcl5 mutant primer**	GGTTCCATCTCGCGCCGC	TTGCGGCTATGACTGAGGAAG
Ucp1	ACTGCCACACCTCCAGTCATT	CTTTGCCTCACTCAGGATTGG
Ucp2	CAGATGTGGTAAAGGTCCGCT	TTCCTCTCGTGCAATGGTCTT
Pgc1α	TCACTCTCAGTAAGGGGCTG	ACCAGAGCAGCACACTCTAT
Cox8b	GAACCATGAAGCCAACGACT	GCGAAGTTCACAGTGGTTCC
Elovl3	TGTTGGCCAGACCTACATGA	ATCTGACTACGGCGTCATCC
Cidea	TGCTCTTCTGTATCGCCCAGT	GCCGTGTTAAGGAATCTGCTG
Ear2	CCTGTAACCCCAGAACTCCA	CAGATGAGCAAAGGTGCAAA
C/ebpβ	GCAAGAGCCGCGACAAG	GGCTCGGGCAGCTGCTT
Cxcr2	TGTCGTCCTTGTCTTCCTG	GGCCTTGTCAATGTCATCG
Tnfα	GGCCTCCCTCTCATCAGTTC	GGTGGTTTGCTACGACGTG
Il-6	GTCCTTCCTACCCCAATTTCCA	TAACGCACTAGGTTTGCCGA
Il-1β	TGCCACCTTTTGACAGTGATG	TAACGCACTAGGTTTGCCGA
F4/80	CTTTGGCTATGGGCTTCCAGTC	GCAAGGAGGACAGAGTTTATCGTG
Mgl-1	TGAGAAAGGCTTTAAGAACTGGG	GACCACCTGTAGTGATGTGGG
Cxcl1	GACCATGGCTGGGATTCACC	CCAAGGGAGCTTCAGGGTCA
Cxcl2	GAAGTCATAGCCACTCTCAAGG	CCTCCTTTCCAGGTCAGTTAGC
Cxcl3	CTGCACCCAGACAGAAGTCAT	CCGTTGGGATGGATCGCTTT
Cxcl5	CCTGCCTGAAGGAAGAGAGAG	GAGCACCAGCTCGGGATATG
Pparγ	CGGTTTCAGAAGTGCCTTG	GGTTCAGCTGGTCGATATCAC
Irs1	TTAGGCAGCAATGAGGGCAA	CGTGAGGTCCTGGTTGTGAA
Irs2	CACAACCTATCGTGGCACCT	AAGGTCTCTGAACTGTGGCG
Pdha1	AAGATGCTTGCCGCTGTATC	ATTTGCAAAATTACGGGAAGC
Mpc1	ATGTCCGGAGCAAGGACTTC	ACAGAGGGCGAAAGTCATCC
Mpc2	AATGGGGATTGGTGTGTGCT	TGACCAAATAAACCCTGTAGCCA
Etfdh	GTGCGACTAACCAAGCTGTC	GGATGAACAGTGTAGTGAGTGG
Ndufa6	CGGTGAAACAAGGACGGGAT	GTGTCCGCTGCTTCCATACT
36b4	GAGGAATCAGATGAGGATATGGGA	AAGCAGGCTGACTTGGTTGC

Table S4. Pathology data at 16- and 60-week-old mice

No.	Tissue	Gross pathology 16weeks		Gross pathology 60weeks	
		WT	Cxcl5 KO	WT	Cxcl5 KO
1	**Skin**	0/3	0/3	0/2	0/2
2	**Mammary gland**	0/3	0/3	0/2	0/2
3	**Salivary gland**	0/3	0/3	0/2	0/2
4	**Thymus**	0/3	0/3	0/2	0/2
5	**Thyroid**	0/3	0/3	0/2	0/2
6	**Liver**	0/3	0/3	0/2	0/2
7	**Gall bladder**	0/3	0/3	0/2	0/2
8	**Spleen**	0/3	0/3	0/2	0/2
9	**Pancreas**	0/3	0/3	0/2	0/2
10	**Stomach**	0/3	0/3	0/2	0/2
11	**Small intestine**	0/3	0/3	0/2	0/2
12	**Large intestine**	0/3	0/3	0/2	0/2
13	**Kidney(left)**	0/3	0/3	0/2	0/2
14	**Kidney(right)**	0/3	0/3	0/2	0/2
15	**Adrenal gland(left)**	0/3	0/3	0/2	0/2
17	**Adrenal gland(right)**	0/3	0/3	0/2	0/2
18	**Testes**	0/3	0/3	0/2	0/2
19	**Urinary bladder**	0/3	0/3	0/2	0/2
20	**Epididymis**	0/3	0/3	0/2	0/2
21	**Prostate**	0/3	0/3	0/2	0/2
22	**Seminal vesicle**	0/3	0/3	1/2	0/2
23	**Skeletal muscle**	0/3	0/3	0/2	0/2
24	**Lymph node**	0/3	0/3	0/2	0/2
25	**Brain**	0/3	0/3	0/2	0/2
26	**Eye with optic nerve**	0/3	0/3	0/2	0/2
27	**Spinal cord**	0/3	0/3	0/2	0/2

Table S5. Pathology data at 16- and 60-week-old mice

Age	Genotype		Parameters		
			body weight	Heart weight	Tibia length
16weeks	WT	Aver.	**31.51**	**114.31**	**18.05**
		± SD	3.15	11.67	0.33
	Cxcl5 KO	Aver.	**30.77**	**118.21**	**18.06**
		± SD	2.82	6.65	0.3
60weeks	Wild	Aver.	**50.19**	**161.07**	**18.61**
		± SD	3.57	10.68	0.28
	Homo	Aver.	**47.49**	**163.6**	**18.62**
		± SD	4.25	7.65	0.26

n=9 (male WT), 9 (male KO)

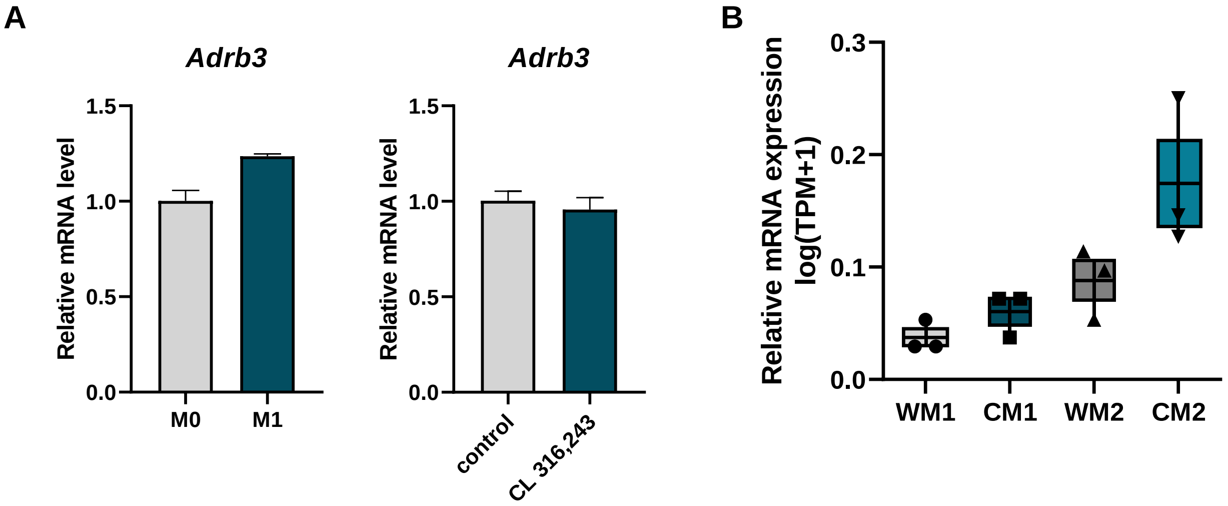

Fig. S1 *Adrb3* is expressed in M1 macrophages. A: *Adrb3* mRNA expression of M0, M1, and CL 316,243-treated M1 macrophages. B: Expression of *Adrb3* in M1 and M2 macrophages of visceral adipose tissue (VAT) RNA-sequencing data during cold exposure. Data are expressed as the mean ± SEM. Statistical analysis was performed using two-tailed unpaired Student's t-tests.

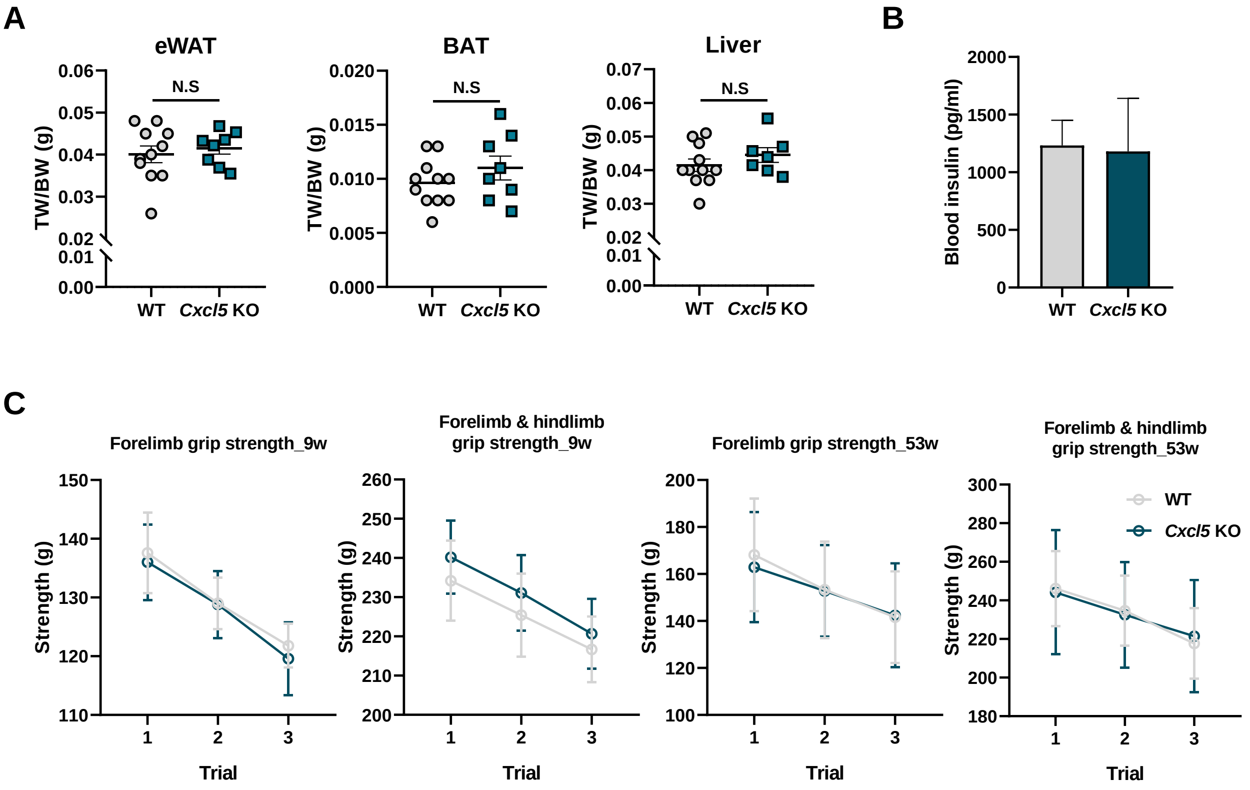

Fig. S2 Tissue weight, blood insulin concentration, and grip strength of *Cxcl5* KO mice A: Epididymal WAT, BAT, and liver weight/body weight of WT and Cxcl5 KO mice. B: Blood insulin concentration in 60-week-old WT (n=9) and Cxcl5 KO mice (n=9) with ELISA analysis C: Grip strength test in 9-week-old WT (n=9) and Cxcl5 KO mice (n=9). Data are expressed as the mean ± SEM. Statistical analysis was performed using two-tailed unpaired Student's t-tests. N.S; not significant.

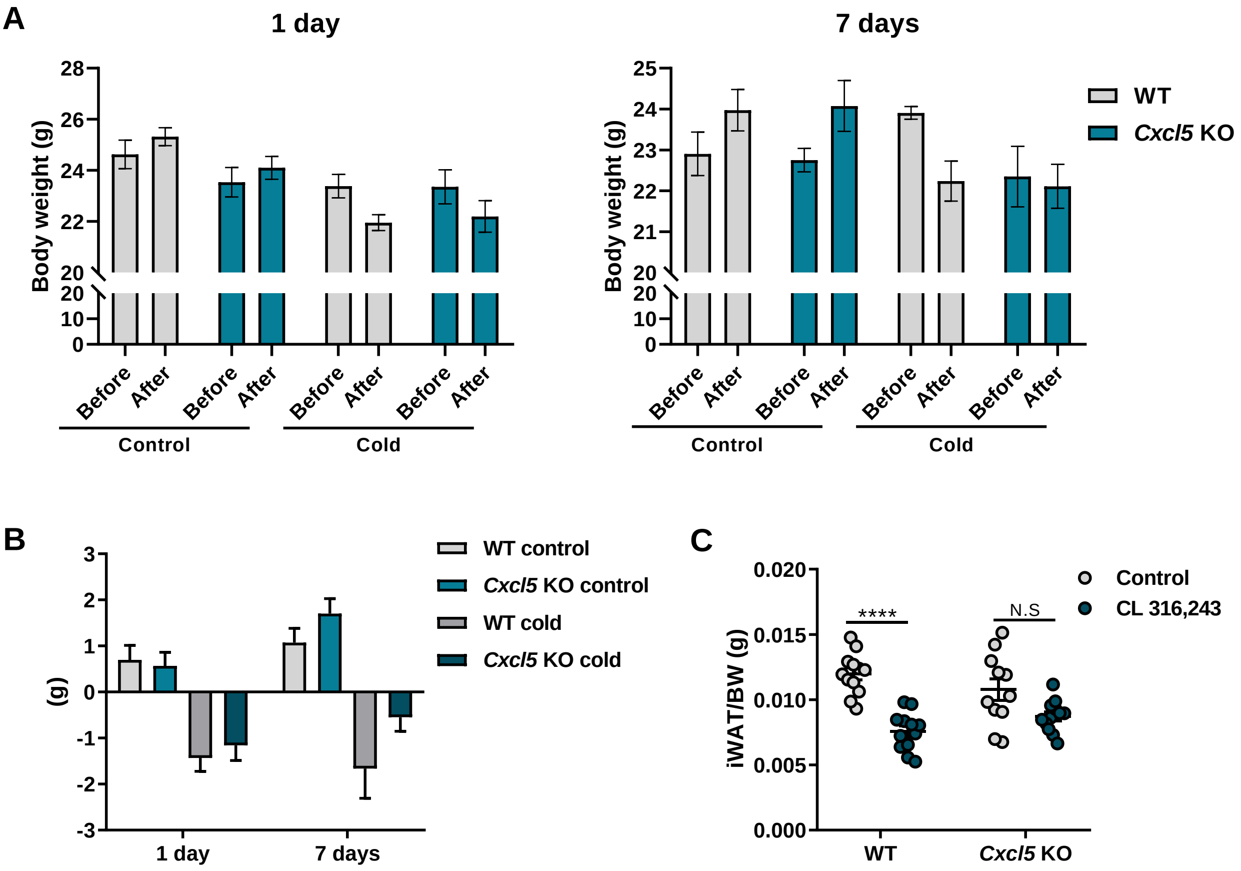

Fig. S3 Body weight change only in WT but not in Cxcl5 KO mice A: Graphs of weight gain during exposure to cold for 1or 7 days. B: Difference in the weight of the mouse before and after exposure to cold. C: iWAT weight normalized by body weight.
